# Synthesis and Characterization of Silica–Tantala Microporous Membranes for Gas Separations Fabricated Using Chemical Vapor Deposition

**DOI:** 10.3390/membranes12090889

**Published:** 2022-09-16

**Authors:** Sean-Thomas B. Lundin, Hongsheng Wang, S. Ted Oyama

**Affiliations:** 1 Department of Chemical System Engineering, The University of Tokyo, 7-3-1 Hongo, Bunkyo-ku, Tokyo 113-8556, Japan; 2 National Institute of Advanced Industrial Science and Technology (AIST), Research Institute of Chemical Process Technology, 4-2-1 Nigatake, Miyagino-ku, Sendai 983-8551, Japan; 3 College of Chemical Engineering, Fuzhou University, Fuzhou 350116, China; 4 Department of Chemical Engineering, Virginia Tech, Blacksburg, VA 24061, USA

**Keywords:** tetraethyl orthosilicate (TEOS), tantalum (V) ethoxide, chemical vapor deposition, microporous silica membrane, gas separation membrane

## Abstract

Composite membranes consisting of microporous tantalum-doped silica layers supported on mesoporous alumina substrates were fabricated using chemical vapor deposition (CVD) in both thermal decomposition and counter-flow oxidative deposition modes. Tetraethyl orthosilicate (TEOS) was used as the silica precursor and tantalum (V) ethoxide (TaEO) as the tantalum source. Amounts of TaEO from 0 mol% to 40 mol% were used in the CVD gas mixture and high H_2_ permeances above 10^−7^ mol m^−2^ s^−1^ Pa^−1^ were obtained for all conditions. Close examination was made of the H_2_/CH_4_ and O_2_/CH_4_ selectivities due to the potential use of these membranes in methane reforming or partial oxidation of methane applications. Increasing deposition temperature correlated with increasing H_2_/CH_4_ selectivity at the expense of O_2_/CH_4_ selectivity, suggesting a need to optimize membrane synthesis for a specific selectivity. Measured at 400 °C, the highest H_2_/CH_4_ selectivity of 530 resulted from thermal CVD at 650 °C, whereas the highest O_2_/CH_4_ selectivity of 6 resulted from thermal CVD at 600 °C. The analysis of the membranes attempted by elemental analysis, X-ray photoelectron spectroscopy, and X-ray absorption near-edge spectroscopy revealed that Ta was undetectable because of instrumental limitations. However, the physical properties of the membranes indicated that the Ta must have been present at least at dopant levels. It was found that the pore size of the resultant membranes increased from 0.35 nm for pure Si to 0.37 nm for a membrane prepared with 40 mol% Ta. Similarly, an increase in Ta in the feed resulted in an increase in O_2_/CH_4_ selectivity at the expense of H_2_/CH_4_ selectivity. Additionally, it resulted in a decrease in hydrothermal stability, with the membranes prepared with higher Ta suffering greater permeance and selectivity declines during 96 h of exposure to 16 mol% H_2_O in Ar at 650 °C.

## 1. Introduction

The delivery and separation of gases are important unit operations in both small- and large-scale chemical processes. Of particular interest are catalytic membrane reactors, where a membrane is used in conjunction with a catalyst bed to combine reaction and mass transfer. Catalytic membrane reactors can reduce the number of required unit operations and increase process efficiency [1].

Membrane reactors can be classified as extractors or distributors [2]. As examples of an extractor, in steam methane reforming, the application of either a hydrogen [3] or a carbon dioxide [4] permeable membrane can simultaneously separate either product into a purified steam and shift the equilibrium conversion of methane forward. As an example of a distributor, in propylene epoxidation with H_2_/O_2_ mixtures, the application of a hydrogen transport membrane can feed hydrogen into the reactor and increase the conversion of propylene while avoiding the explosive regime [5]. Other examples of distributed feed reactors include the use of an oxygen-selective membrane to reduce the oxygen partial pressure and avoid the overoxidation of products in the partial oxidation of methane to syngas (POM) [6,7], the oxidative coupling of methane (OCM) [8,9], and the partial oxidation of methane to formaldehyde [10,11]. Notably, however, a nonselective porous MgO membrane for OCM showed no improvement in performance over a packed-bed reactor due to the backpermeation of non-O_2_ gas species [12], which suggests that the permselectivity of the desired permeation gas is critical to membrane reactor performance.

Membranes for gas separations include a variety of materials, including dense metal membranes [13], polymeric membranes [14], and ceramic membranes [15]. Among the ceramic membrane materials, microporous silica-based membranes have garnered significant interest primarily due to their high H_2_ permeance and high permselectivity over larger gas molecules [16,17]. The benefits of using microporous silica-based membranes include a low materials cost, along with a high thermal stability and chemical resistance [18].

**Figure 1:**
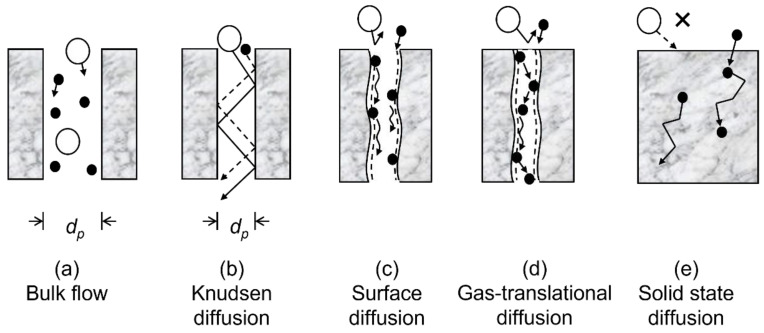
Schematic of gas separation mechanisms.

Silica-based membranes have a research history spanning more than 30 years since the first reports by Gavalas et al. [19] and Okubo and Inoue [20] in 1989. Typical synthesis techniques include chemical vapor deposition (CVD) [21] or sol–gel [22] methods to form separation layers less than 100 nm thick on mesoporous substrates [23]. The permeation of gases through silica-based membranes can involve several mechanisms, which are shown in Figure 1 and described in detail elsewhere [24]. Bulk flow is unselective and occurs when the pore diameter, *d_p_*, is much larger than the mean free path of gas molecules. Knudsen diffusion results in selective flow that scales with the inverse ratio of the square root of the molecular masses and occurs when the pore diameter is similar to the mean free path. Surface diffusion can result in high selectivity and occurs when surface modifications allow a specific gas to adsorb and diffuse more quickly than gas flow would allow. However, these mechanisms do not control selectivity in microporous membranes. Instead, highly permselective microporous silica membranes rely primarily on solid-state diffusion for the smallest gases (H_2_, He, Ne), and gas-translational diffusion for slightly larger gases (N_2_, CH_4_, CO, CO_2_) [24,25]. For the smaller gas species, pore size is not important because permeance occurs by jumps between adjacent cavities in the silica structure (called solubility sites), and applications have been reported such as hydrogen purification (H_2_/CH_4_) [23] and helium recovery (He/N_2_) [26]. For the larger species, pore size is important and other types of uses have been published such as carbon capture (CO_2_/CH_4_) [27] and olefin/paraffin separations (C_3_H_6_/C_3_H_8_) [28].

One issue that continues to limit the application and utilization of silica-based membranes is the instability of silica in the presence of steam. The instability arises from the densification of the porous silica network due to the formation of hydroxyls and the hydrolysis of Si-O-Si linkages [29,30]. Attempts to increase the hydrothermal stability have involved the addition of secondary elements in an effort to stabilize the silica matrix. Alumina [30], zirconia [31], cobalt [32], nickel [33], titania [34], niobia [35], tantala [36], and magnesia [37] additions have been studied, as well as methyl group incorporation [25,38,39], and their effect on the performance of silica-based composite membranes upon exposure to steam is summarized in Table 1. From these data, it appears that exposure to H_2_O causes a loss of H_2_ permeance for all membrane combinations, with declines ranging from 25% to 87%. Notably, early group transition elements (Ti, Nb, and Ta), appear to result in less H_2_ permeance loss than later transition elements (Co, Ni, Fe). However, Zr shows conflicting data from several studies.

Though additional elements influence the hydrothermal stability of silica-based composite membranes, the performance varies between additives. Some elements, such as Al and Zr, have been shown to increase hydrothermal stability, but other elements, such as Mg, have been shown to increase the mobility of silica and lead to sintering and densification [37]. Thus, to enhance the hydrothermal stability of silica-based composite membranes, additive elements must be carefully selected and tested for efficacy.

Studies using Nb or Ta precursors are limited, but some reports have suggested that these metal additions can affect the silica network pore size and resultant hydrothermal stability. Qureshi et al. [44] reported that membranes formed using a sol–gel process by adding Nb or Ta precursors to bis-triethoxysilylethane (BTESE) resulted in higher permeance and a more open pore structure than membranes formed with BTESE alone. However, hydrothermal stability was not measured. A recent study [36] reported that a membrane formed from TEOS and TaEO showed excellent selectivity and hydrothermal stability. Boffa et al. [35] added Nb to a silica membrane and observed an increase in hydrothermal stability, but the permeance was significantly lower than a silica control membrane, which may have affected the result. Thus, further investigations should be made to understand the changes in pore size and hydrothermal stability of Ta addition.

The present paper describes the synthesis and characterization of a silica–tantala composite membrane, prepared by mixed-precursor CVD. The permeances of He, H_2_, N_2_, O_2_, and CH_4_ were measured for membranes fabricated with various ratios of Ta/Si, and the hydrothermal stability performance was systematically discussed and analyzed. The specific selectivities of H_2_/CH_4_ and O_2_/CH_4_ were monitored due to their relevance to membrane reactor applications in methane reforming and partial oxidation of methane.

## 2. Materials and Methods

### 2.1. Membrane Synthesis

Membranes were synthesized via a two-step procedure involving the preparation of a γ-alumina intermediate layer by a sol–gel procedure followed by chemical vapor deposition (CVD) of the microporous silica membrane onto a porous α-alumina support [30,34,38,42]. Initially, a small section of an asymmetric porous α-alumina support (Noritake Co., I.D. = 7 mm, O.D. = 10 mm, length = 15 mm, nominal pore size = 60 nm) was sealed onto two nonporous alumina tubes (Sakaguchi E.H Voc Co., Tokyo, Japan, I.D. = 7 nm, O.D. = 10 mm, length = 200 mm) using a glass paste (GA-13/325, Nippon Electric Glass Co., Ltd., Shiga, Japan) mixed with terpineol (Nisshin Kaisei Co., Ltd., Saitama, Japan) and fired at 1000 °C for 10 min with a heating and cooling rate of 5 °C min^−1^.

The sealed α-alumina support then had a γ-alumina intermediate layer applied via a sol–gel process to further reduce the 60 nm pore size. This was done by creating boehmite sols of 80 nm and 40 nm average particle sizes, followed by successive dip-coating and firing of the support in the larger and smaller boehmite sol. After immersing the support in one of the sols for 10 s, the membrane support was slowly withdrawn from the solution and dried in a cleanroom for 4 h at room temperature before being calcined at 650 °C for 3 h using a heating and cooling rate of 1.5 °C min^−1^.

The 80 nm and 40 nm boehmite sols were prepared by mixing 0.3 mol of aluminum isopropoxide (Aldrich, >98%) in 50 mL of distilled water and stirring for 24 h at 98 °C. Afterwards, nitric acid (Wako, 60%) was slowly added (80 nm, H^+^/Al = 0.025; 40 nm, H^+^/Al = 0.070) and mixed for a further 24 h at 98 °C to induce peptization (oligomerization). To enhance the colloidal stability of the boehmite sol, a solution of 0.7 g polyvinyl alcohol (Polyscience, M.W. = ca. 78,000) in 20 mL of distilled water was added. Finally, the total volume was adjusted to 200 mL by adding distilled water and stirring at 70 °C for 3 h. The particle size distributions were determined by a dynamic light scattering analyzer (Horiba LB-550, Japan) and confirmed to have averages of 40 nm and 80 nm.

Once the γ-alumina intermediate layer was applied and calcined, the microporous silica–tantalum membrane was prepared by CVD using tetraethyl orthosilicate (TEOS, Aldrich, 98%) as the silica precursor and tantalum (V) ethoxide (TaEO, Aldrich, 99.98%) as the tantalum precursor. Use was made of a CVD apparatus detailed earlier [42] to deliver the TEOS and TaEO. The vapor pressure of each precursor was used to calculate the ratio of TaEO to TEOS in the CVD reactor. The vapor pressure of TEOS was obtained from the Antoine equation

(1) $$\log p=A-\frac{B}{T+C}$$

where p is the partial pressure of the component, T is the temperature in Kelvin, and the coefficients are: A=4.17312, B=1561.277, and C=−67.572. The vapor pressure of TaEO was calculated via the Claussius–Clapeyron relation

(2) $$\ln\frac{p}{p_{ref}}=\frac{\Delta H_{vap}}{R}\left(\frac{1}{T_{ref}}-\frac{1}{T}\right)$$

where $\Delta H_{vap}$ is the enthalpy of vaporization (79.1 kJ mol^−1^), R is the ideal gas constant, and $p_{ref}$ is a reference partial pressure of TaEO ($p_{ref}=0.0133\;\mathrm{kPa}$ at 145 °C).

A summary of the synthesized membranes and relevant CVD parameters is given in Table 2. The nomenclature reflects the Si–Ta composition and the CVD temperature. All membranes were synthesized at 101 kPa. Synthesis of the silica control membrane (Si-650) consisted of flowing 5 mL min^−1^ (3.7 µmol s^−1^) of Ar through the TEOS bubbler and diluting with 15 mL min^−1^ (12 µmol s^−1^) of Ar. All flow rates are here in ml min^−1^ at normal conditions (25 °C, 101 kPa) and may be converted to µmol s^−1^ by division by 1.5. The flow of TEOS was kept constant, equivalent to 9.2 nmol s^−1^ of Si, for all synthesis conditions and the 15 mL min^−1^ Ar dilution stream was diverted through the TaEO bubbler as appropriate. Thus, the total flow into the reactor was constant at 20 mL min^−1^ (15 µmol s^−1^). In this manner, the TaEO/TEOS ratio was controlled solely by the temperature of the TaEO bubbler, which ranged from 117 °C to 173 °C. The sweep side of the membrane was maintained at a total flow equal to the feed stream containing the precursors (20 mL min^−1^). Ar was used for the higher deposition temperatures of 600 °C and 650 °C; however, Ar was changed to O_2_ for the lower deposition temperatures of 400 °C and 500 °C to enhance the deposition rate by counter-flow reactive CVD.

The CVD process was interrupted periodically to check the progress of deposition and membrane fabrication by measuring pure gas permeation properties in the same apparatus as the synthesis. Gases used in this study included Ar (99.99%), He (99.999%), H_2_ (99.99%), O_2_ (99.5%), N_2_ (99.99%), and CH_4_ (99.99%). Permeation measurements were conducted by flushing the feed with the gas of interest for several minutes before pressurizing to a transmembrane pressure of 0.2 MPa; the permeate was at atmospheric pressure (0.1 MPa). A digital flowmeter (GF1010, GL Science) was used to measure permeation flow rates above 1 mL min^−1^, whereas permeation flow rates below this were measured by carrying the permeate using a 50 mL min^−1^ Ar sweep flow to a gas chromatograph equipped with a thermal conductivity detector (GC-TCD). The GC-TCD (490 Micro GC, Agilent Technologies) was installed with a molecular sieve 5A column capable of detecting He, H_2_, N_2_, O_2_, and CH_4_.

### 2.2. Membrane Characterization

The characterization of the membranes was performed after permeance testing by fracturing the membrane tube to expose the inner surface (CVD deposition side). The samples were stored in air between testing and characterization. Surface and cross-sectional imaging was conducted without pretreatment using a Hitachi SU8020 scanning electron microscope (SEM) at an accelerating voltage of 15 kV, and elemental analysis was performed via an equipped energy dispersive X-ray spectroscopy (EDS, Horiba, Kyoto, Japan) attachment. X-ray photoelectron spectroscopy (XPS) was performed with a PHI 5000 VersaProbe (Ulvac-Phi Inc., Kanagawa, Japan) equipped with a monochromatized Al Kα X-ray source (1486.6 eV). Narrow scans of the elements of choice (C1s, O1s, Al2p, Si2p, Ta4f, and Ta4d) were conducted with a pass energy of 23.5 eV, energy per step of 0.200 eV, and time per step of 50 ms. The X-ray source was set to a 100 µm focus size, 25 W power, and 15 kV voltage for all scans. To verify carbon was not incorporated into the silica structure, adventitious surface carbon was removed with a 30 s Ar sputter using a 1 kV2 × 2 setting (ca. 2 nm min^−1^ removal of SiO_2_). The depth profile XPS of membrane Si-650 used the higher Ar sputter setting of 4 kV2 × 2 (ca. 25 nm min^−1^ removal of SiO_2_). Collected spectra were analyzed using the MultiPak software suite.

## 3. Results and Discussion

### 3.1. Microstructure Analysis

**Figure 2:**
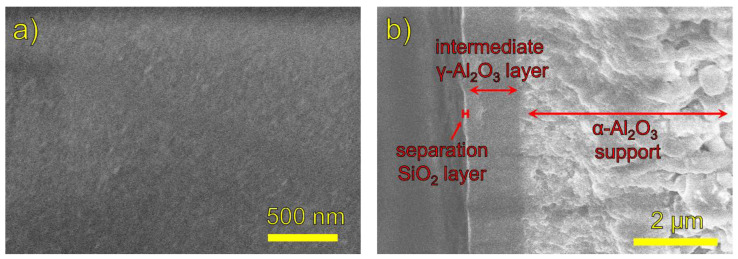
SEM micrographs of membrane Si-10Ta-650: (**a**) surface image; (**b**) cross-sectional image.

The microstructure of the synthesized membranes was probed using SEM. As an example, Figure 2 shows the surface and cross-sectional images of the Si-10Ta-650 membrane. The surface image (Figure 2a) shows a uniform, grainy texture with no defects. The cross-sectional image (Figure 2b) shows the porous alumina support as a light gray region on the right, and the sol–gel-fabricated alumina layer as a darker gray region spanning about 1.3 µm. The thin bright region on the left is the Si–Ta CVD layer, which is estimated to have a thickness on the order of 10 nm. A series of surface images for each membrane can be found in Figure S1 of the Supplementary Materials.

### 3.2. Elemental Analysis

Attempts were made to obtain the elemental composition of the membranes by EDS, XPS, and XANES measurements. Although the Si and Al contents were determined, the Ta levels could not be obtained (Figure S2) by any technique. This is not surprising. For EDS, the Ta M-line and the Si K-lines overlap exactly [45]. For XPS and XANES, the Ta Lα1 line at 8.145 keV has an electron escape depth of ca. 5 nm [46] and this is not sufficient for bulk analysis. Information on the samples measured and the data collected can be found in the Supplementary Materials Section S2. XPS and EDS measurements were conducted to quantify Si and Al in the membrane materials, and the results are presented in Table 3, with the raw data for XPS listed in Table S1. To remove the effects of adventitious carbon, a 30 s Ar sputter was conducted within the XPS. Again, the analytical methods used were not able to detect Ta because of instrumental limitations. Its presence, at least at the level of a dopant, was inferred from the systematic changes it imparted on the permeance properties of the membranes. It has been stated that the nominal sensitivity of XPS is about 0.1 at.%. However, it has also been noted that the elemental sensitivity factors for various elements can differ by as much as a factor of 100 [47]. Thus, the maximum levels of Ta in the samples studied here were estimated to be 0.1–10 at.%. As is shown in later sections, increasing Ta in the membranes resulted in a slight increase in pore size and a decrease in hydrothermal stability.

Interestingly, a comparison of the elemental analysis by XPS and EDS showed opposing trends. The XPS results found no Al detected for the Si-650 and Si-3Ta-650 membranes; however, at higher concentrations of Ta in the CVD mixture, the presence of Al was indicated. The EDS measurements, by comparison, showed a low Si content for the Si-650 with the Si content growing with increasing Ta in the CVD mixture. One possibility is that the presence of TaEO in the mixture impeded the deposition even though the Ta was not greatly incorporated. This was indicated by the longer CVD time required to form the same closed pore structure when Ta was present.

**Figure 3:**
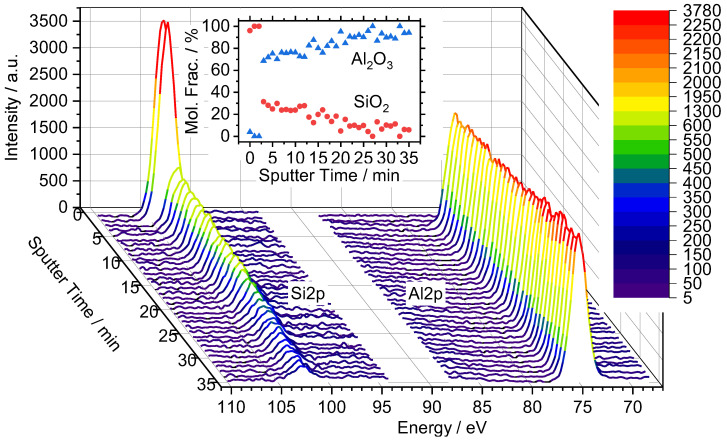
Si2p and Al2p XPS peaks of membrane Si-650 throughout the Ar surface sputter. Inset shows Al_2_O_3_ and SiO_2_ mole fractions vs. sputter time.

For the depth profile analysis, the Si-650 membrane was selected due to the initial XPS compositional analysis not detecting any Al in the sample. It should be noted that the sputtering rates of Si and Al are the same, while that of Ta is about 5% higher [48]. Although the XPS penetration depth is small, the lack of Al signal suggests that the Si-650 membrane had the thickest silica layer deposited by CVD. This is consistent with the observation, discussed in the next section, that the addition of TaEO to the CVD mixture slowed the permeation decline and led to a larger pore network formation. Figure 3 shows the Si2p and Al2p peaks as a function of sputter time for Si-650. The first 3 min of sputter time detected nearly zero Al peak intensity, and with a sputter rate of about 25 nm min^−1^ the thickness of the top layer of silica was estimated to be less than 100 nm. After this period, the Al peak intensity continued to rise while the Si peak intensity fell. The Si2p peak intensity was still detectable after 35 min but was quite small. The CVD gases likely infiltrated the alumina pore network during early deposition periods, leading to a small amount of penetration and deposition of Si in the support. Although the silica may have deposited deep into the support, the selective silica layer was likely near the top surface where Al was not detected. This analysis provides an indication of the separation portion of the silica layer thickness and is supported by SEM images of microporous silica layers from the authors’ previous studies being less than 100 nm [40,42].

### 3.3. Effect of Increasing Tantalum Content

**Figure 4:**
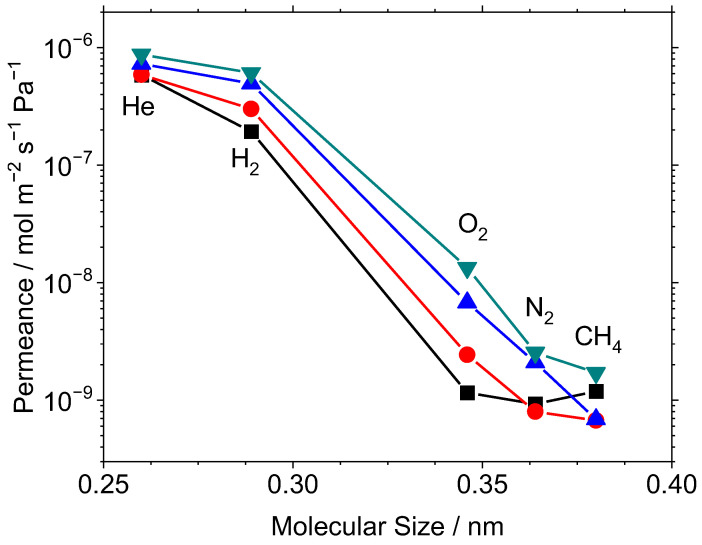
Permeance of several gases at 650 °C after CVD for various Si/Ta precursor ratios: Si-650 [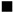], Si-3Ta-650 [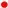], Si-10Ta-650 [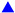], Si-40Ta-650 [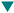].

Increasing TaEO content in the CVD gas mixture at 650 °C resulted in an increase in gas permeance for most gases tested; the gas permeance for several gases measured at 400 °C is shown in Figure 4. Additionally, all three membranes with tantalum ethoxide in the CVD mixture (Si-3Ta-650, Si-10Ta-650, Si-40Ta-650) resulted in separation factors that appeared to be based purely on the size of the gas molecules. Unfortunately, Ne was not measured so the likely site-hopping mechanism [21,30,34] could not be established. Nevertheless, for the larger gas species the synthesis resulted in good pore size control during membrane synthesis and an effective molecular sieving structure. Only in the case of the Si-650 membrane was the CH_4_ permeance higher than the N_2_ permeance, suggesting defects in the pore size may have resulted in Knudsen flow that adversely affected selectivity. In this case, the permeances of the larger molecules appeared to be between Knudsen and molecular sieving, with O_2_ and CH_4_ permeances being nearly equal. The flow of small molecules (He, H_2_) appeared unaffected because the contribution of Knudsen flow from defects was expected to be less than 2% of the H_2_ permeance (e.g., assuming 100% Knudsen regime for CH_4_ permeance would result in H_2_ permeance of only 3.4 × 10^−9^ mol m^−2^ s^−1^ Pa^−1^, or 1.7% of total H_2_ permeance). In fact, the He and H_2_ were likely permeating by a solid-state site jump mechanism [21,30,34] through the dense part of the membrane, which actually was a network of solubility sites.

**Figure 5:**
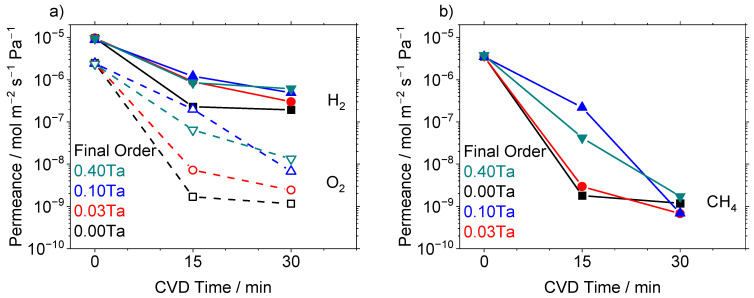
(**a**) Permeance of H_2_ and O_2_ vs. CVD time at 650 °C for the various Si/Ta precursor ratios. (**b**) Permeance of CH_4_ vs CVD time at 650 °C for the various Si/Ta precursor ratios. Legend: Si-650 [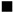], Si-3Ta-650 [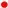], Si-10Ta-650 [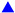], Si-40Ta-650 [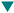].

One potential explanation for the increase in pore size control and enhancement in selectivity for O_2_, N_2_ and CH_4_ gases is that the addition of TaEO resulted in a slower deposition rate. As Figure 5 shows, the decline in gas permeance versus CVD time was slower with increasing TaEO levels. This slower decline in gas permeation may have been caused either by a slower deposition rate leading to a slower pore closure or by interactions of TaEO with TEOS to form larger pores during the deposition process. The addition of secondary precursors to the CVD mixture has been shown previously to slow the pore closure rate [42].

### 3.4. Thermal vs. Reactive Deposition

**Figure 6:**
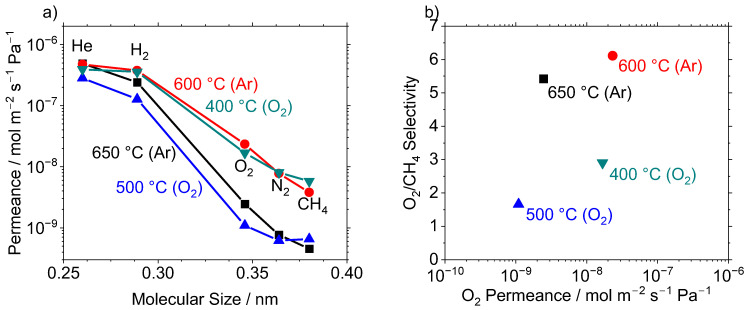
(**a**) Permeance of several gases measured at 400 °C after CVD of Si-10Ta membranes at the denoted temperatures for thermal (Ar) and reactive (O_2_) CVD conditions. (**b**) O_2_/CH_4_ selectivity vs. O_2_ permeance at 400 °C for various synthesis conditions of Si-10Ta membranes. Legend: Si-10Ta-650 [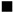], Si-10Ta-600 [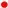], Si-10Ta-500 [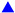], Si-10Ta-400 [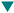].

To better understand the deposition behavior, CVD was conducted under both thermal deposition conditions involving only Ar gas and reactive deposition conditions using O_2_ gas (Figure 6). Because the Si-10Ta-650 membrane showed the highest selectivities (H_2_/CH_4_ of 720, O_2_/CH_4_ of 10 measured at 650 °C), the TaEO/TEOS ratio of 0.10 was used for these depositions. The O_2_ oxidized TEOS and TaEO, which significantly reduced the deposition temperature of the precursors, and a membrane was successfully deposited at 400 °C. A lower temperature of 350 °C was attempted but was unsuccessful in forming a selective membrane even after 6 h of deposition. Although it is reasonable to assume that a membrane would form after extremely long CVD times [49,50], this study focused on deposition times of within a few hours to keep the process manageable.

### 3.5. Hydrothermal Stability Tests

**Figure 7:**
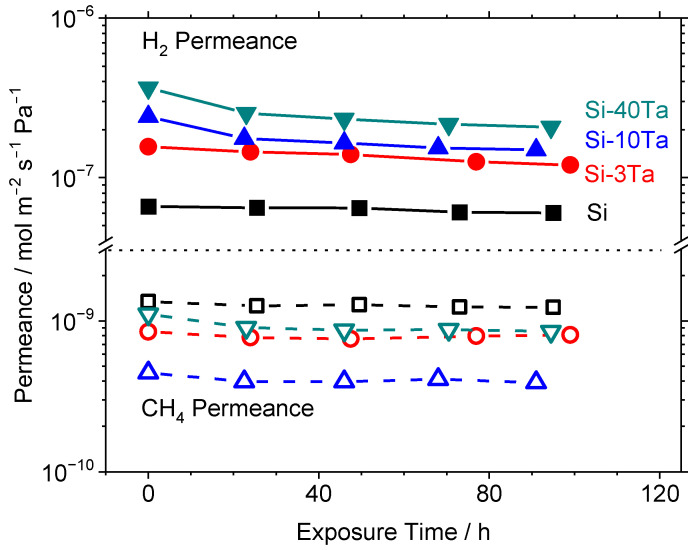
Pure gas permeance of H_2_ and CH_4_ vs. exposure time at 650 °C to 16 mol% H_2_O in Ar. Si-650 [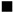], Si-3Ta-650 [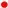], Si-10Ta-650 [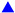], Si-40Ta-650 [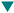]. Permeance measurements taken during short interruptions of the steam exposure.

Previous studies have shown that the addition of secondary elements, such as Al [30], Co [32], or Nb [35] to the silica network can improve hydrothermal stability. Thus, a series of hydrothermal stability tests was performed on the Si–Ta membranes to determine if Ta had any beneficial effects. Figure 7 shows the results for a series of Si–Ta membranes exposed to 16 mol% H_2_O in Ar at 650 °C for 100 h. These pure gas measurements were taken by interrupting the steam exposure and purging with Ar to prevent any effects of adsorbed H_2_O. As previously mentioned, the addition of TaEO increased the gas permeances, but this appeared to have a negative impact on the hydrothermal stability. While Si-650 experienced a 9% decline in both H_2_ and CH_4_ permeances, the Si-40Ta-650 membrane experienced a decline of 43% in H_2_ permeance and a decline of 23% in CH_4_ permeance. This trend means that the addition of TaEO caused an overall loss of ideal selectivity compared to the pure TEOS membrane.

**Figure 8:**
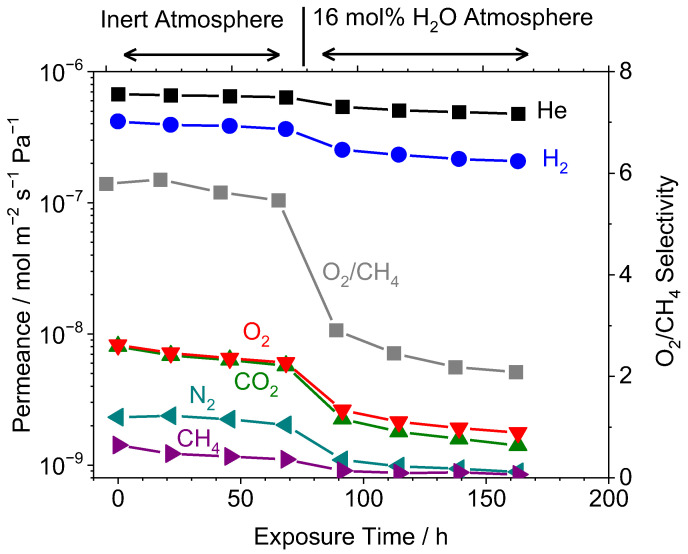
Pure gas permeance of various gases at 650 °C versus time of exposure to either Ar (0–69 h) or 16 mol% H_2_O in Ar (69–163 h) for the Si-40Ta-650 membrane. Permeance measurements taken during short interruptions of the steam exposure.

Interestingly, the largest flux decline occurred for midsized gases (O_2_, CO_2_, N_2_, CH_4_), rather than the smaller gases (He, H_2_). This is because the larger gases permeate through the pores, which are eliminated by the hydrothermal treatment, while the smaller gases permeate by solid-state diffusion, which is unaffected by densification. Figure 8 shows the permeances of all gases measured on Si-40Ta-650 during both the initial thermal stability region (0–69 h) and the hydrothermal exposure (69–163 h). During the initial stabilization period under Ar gas (inert atmosphere), the H_2_, O_2_, and CH_4_ permeances declined by 13%, 26%, and 22%, respectively. Despite the smaller kinetic diameter of CO_2_ (0.33 nm) compared to O_2_ (0.346 nm), the CO_2_ permeance initial and final measurements were nearly identical to O_2_. This indicates that polar effects were present and the CO_2_ permeance was inhibited by adsorption in the pores, which has been explored by other groups [43,51]. More notable is that the permeance declines for this Si–Ta membrane were more severe than the loss that the TEOS membrane (Si-650) experienced during the hydrothermal stability treatment, suggesting that the addition of TaEO created a more unstable pore structure. This is consistent with a larger pore structure as the driving force for densification and pore closure. Under steam, the gas permeances were lowered even more severely than the selectivities, with H_2_, O_2_, and CH_4_ permeances declining by 57%, 69%, and 23%, respectively.

Previous studies have resulted in claims that the addition of auxiliary elements can increase the hydrothermal stability, but it is interesting to note that occasionally the increase in stability occurs for the membranes with lower initial permeance. For instance, the initial H_2_ permeances of the pure SiO_2_ membranes were higher than the SiO_2_-Al_2_O_3_ membrane in the work of Amanipour et al. [52] and the SiO_2_-TiO_2_ membrane in the work of Gu and Oyama [34], but both studies reported a significant decline in H_2_ permeance for the SiO_2_ membrane. In those studies, the decline was so severe that the final H_2_ permeance of the SiO_2_ membrane was lower than that of the mixed element membranes, which supports the claim that these auxiliary elements increased hydrothermal stability. In a more extreme example, the work of Boffa et al. [35] showed an increase in stability for Si-Nb membranes, but the Si-Nb membranes had H_2_ permeance nearly an order of magnitude lower than the SiO_2_ membrane both before and after hydrothermal exposure. Thus, the increase in stability did not result in an increase in H_2_ separation productivity. For comparison to the current work, the most similar study is that of Igi et al. [32], who observed that Co-doped silica membranes resulted in higher initial H_2_ permeance, but also greater rates of decline in performance as compared to a pure silica membrane. Despite the increased rate of decline, however, the decline was slow enough that the stabilized H_2_ permeance remained higher for the Co-doped membrane than for the pure silica after hydrothermal exposure. In the current study, the decline in permeance was more severe for increasing Ta content, but the final H_2_ permeance remained higher for membranes fabricated with a higher Ta content. Thus, the data suggest a lack of hydrothermal stability in Si–Ta membranes, but part of this is due to differences in the initial permeances and selectivities.

The permeance changes as a result of steam exposure were compared to changes in the effective pore size of the membranes. Measuring the pore size of molecular sieve membranes is a challenge because of the sub-nanometer sizes. The bubble-point method is limited to about 50 nm due to the increasing pressure required, while N_2_ adsorption measurements are limited to pore sizes above the kinetic diameter of N_2_ (0.364 nm). The only physical measurement technique reported for kinetic sieving level porosity is the positron annihilation lifetime spectroscopy (PALS) technique [53], but this requires sophisticated equipment not commonly available. Instead, a theoretically derived solution known as the normalized Knudsen based permeance equation was used, which assumes the same apparent activation energy of permeation for each gas [54]. The average pore size is related by an expression known as the normalized Knudsen based permeance, *f*,

(3) $$f=\frac{P_{i}}{P_{He}\sqrt{M_{He}/M_{i}}}={\left(\frac{1-d_{i}/d_{p}}{1-d_{He}/d_{p}}\right)}^{3}$$

where $P_{i}$ is the permeance of gas species i, $M_{i}$ is the mass of gas species i, $d_{i}$ is the kinetic diameter of gas species i, and $d_{p}$ is the average pore size. In this equation, *f* represents the deviation from the Knudsen regime due to the permeance contribution from molecular sieving (*f* = 1 for Knudsen flow and is reduced to less than one when molecular sieving contributes to the overall flow).

**Figure 9:**
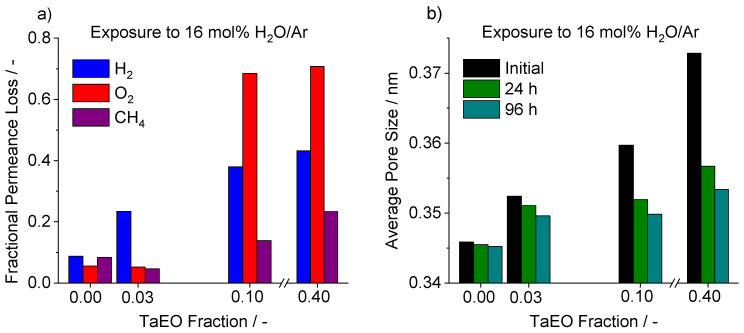
(**a**) Fractional permeance loss at 650 °C of H_2_, O_2_ and CH_4_ for the various Si–Ta membranes after exposure to a 16 mol% H_2_O in Ar gas atmosphere for 96 h. (**b**) Change in calculated pore size of the Si–Ta membranes from exposure to a 16 mol% H_2_O in Ar stream after 24 h and 96 h.

The decrease in permeance of gases is summarized in Figure 9a, and can be compared to the change in average pore size in Figure 9b. The addition of TaEO increased the resultant initial pore size of the membranes, and a clear relationship between a higher initial pore size and a greater decline under hydrothermal exposure is evident; in fact, Si-650 had no TaEO content and saw only a small change in the gas permeances and pore size. However, it can be observed that the final pore size remained larger with increasing TaEO content.

Figure 9b shows that the average pore size tends to increase with TaEO fraction. Matching this, the permeance of H_2_ also increases. However, the permeances of O_2_ and CH_4_ deviate from this trend, indicating that the pore size is not a factor in their permeance except at the highest pore sizes.

## 4. Conclusions

Various conditions for the preparation of tantalum-doped microporous silica membranes by CVD were explored. The concentration of tantalum (V) ethoxide added to tetraethyl orthosilicate was varied from 0 to 40 mol% to form membranes at deposition temperatures of 400 °C to 650 °C, using either thermal decomposition or oxidative-assisted decomposition. High H_2_ permeances in the range of 10^−7^ mol m^−2^ s^−1^ Pa^−1^ were obtained, with permeance increasing with Ta content in the CVD mixture. A 10% Ta mixture produced the best mix of H_2_ permeance (4.9 × 10^−7^ mol m^−2^ s^−1^ Pa^−1^) and selectivity (H_2_/CH_4_ of 720, O_2_/CH_4_ of 10) measured at 650 °C and was selected as the base case to test with other synthesis parameters. Variations in temperature showed an increase in H_2_/CH_4_ selectivity at higher deposition temperatures, at the expense of O_2_/CH_4_ selectivity. Measured at 400 °C, the highest H_2_/CH_4_ selectivity of 530 was obtained with a membrane prepared using thermal CVD conditions at 650 °C, but the highest O_2_/CH_4_ selectivity of 6 was obtained by lowering the thermal CVD conditions to 600 °C. Calculation of the resultant pore sizes showed that the average pore size in the membranes after CVD increased from 0.35 nm for pure Si to 0.37 nm for a Si-40Ta CVD mixture. This increase in pore size correlated with an increase in O_2_/CH_4_ selectivity at the expense of H_2_/CH_4_ selectivity due to a shift in pore size around that of the O_2_ molecule (0.346 nm). A slight increase to 0.37 nm created a network of pores larger than O_2_ on average, but still smaller than that of CH_4_ (0.38 nm). This resulted in an outsized increase in O_2_ permeance over that of CH_4_. However, any increase in pore size increased CH_4_ permeation, while having a minimal effect on the solid-state diffusion of H_2_. Additionally, an increase in pore size was correlated with a decrease in hydrothermal stability, with the higher tantalum content membranes suffering greater performance declines during 96 h of exposure to 16 mol% H_2_O in Ar at 650 °C. The decrease in hydrothermal stability is suggested to be caused by a greater tendency for larger pores to densify under the presence of steam.

## Figures and Tables

**Table 1:** Hydrothermal stability of various silica-based composite membranes.

Membrane Material	Synthesis		H_2_O Exposure			H_2_ Permeance		Ref.
	Precursors *	Method	Temp. /°C	H_2_O Conc. /mol %	Time /h	Change /%	Final /mol m^−2^ s^−1^ Pa^−1^	
SiO_2_+Al_2_O_3_	TEOS+ATSB	CVD	650	16	100	−45	3.9 × 10^−8^	[40]
			650	16	96	−68	2.3 × 10^−8^	[40]
			600	16	200	−39	1.3 × 10^−7^	[30]
SiO_2_+ZrO_2_	TEOS+ZTB	Sol–gel	500	50	30	−18	2.8 × 10^−6^	[31]
		Sol–gel	500	13–33	30	−73	1.1 × 10^−7^	[41]
		CVD	650	16	48	−56	1.7 × 10^−7^	[42]
SiO_2_+CO_3_O_4_	TEOS+ Co(NO_3_)_2_·6H_2_O	Sol–gel	500	50	60	−50	4.0 × 10^−8^	[32]
			500	30	60	−47	1.8 × 10^−7^	[43]
SiO_2_+NiO	TEOS+ Ni(NO_3_)_2_·6H_2_O	Sol–gel	40	4.4	1680	−50	3.4 × 10^−7^	[33]
SiO_2_+Fe_2_O_3_	TEOS+ Fe(NO_3_)_3_·9H_2_O	Sol–gel	40	4.4	840	−87	9.3 × 10^−7^	[33]
SiO_2_+TiO_2_	TEOS+TIP	CVD	650	75	125	−30	9.1 × 10^−8^	[34]
SiO_2_+Nb_2_O_5_	TEOS+NPB	Sol–gel	200	56	70	−32	2.6 × 10^−8^	[35]
SiO_2_+Ta_2_O_5_	TEOS+TaEO	CVD	650	16	200	−25	3.5 × 10^−8^	[36]

* TEOS: tetraethyl orthosilicate, ATSB: aluminum tri-sec-butoxide, ZTB: zirconium tert-butoxide, TIP: titanium isopropoxide, NPB: niobium penta(*n*-butoxide), TaEO: tantalum ethoxide.

**Table 2:** CVD synthesis parameters. Ar flow rates through the bubblers were constant at 3.7 µmol s^−1^ through TEOS (providing 9.2 nmol s^−1^ TEOS) and 12 µmol s^−1^ through TaEO.

Membrane ID	Ta/(Si+Ta) Fraction	TaEO Bubbler		Counter Flow Gas	Reactor Temperature	CVD Time /min
		Temperature	Flowrate			
		/°C	/nmol s^−1^		/°C	
Si-650	-	-	-	Ar	650	30
Si-3Ta-650	0.03	117	0.29	Ar	650	30
Si-10Ta-650	0.10	138	0.99	Ar	650	30
Si-40Ta-650	0.40	173	6.1	Ar	650	30
Si-10Ta-600	0.10	138	0.99	Ar	600	45
Si-10Ta-500	0.10	138	0.99	O_2_	500	30
Si-10Ta-400	0.10	138	0.99	O_2_	400	105

**Table 3:** Atomic concentrations from XPS analysis before and after a 30 s Ar sputter, and EDS of the surface.

	XPS						EDS		
Membrane ID	Elemental Fraction/%						Elemental Fraction/%		
	Before Sputtering			After Sputtering					
	O1s	Al2p	Si2p	O1s	Al2p	Si2p	O	Al	Si
Si-650	65	0.0	35	68	0.0	32	73	26	0.5
Si-3Ta-650	68	0.0	32	67	1.5	32	81	14	4.2
Si-10Ta-650	70	13	17	68	18	15	79	14	7.0
Si-40Ta-650	66	4.6	30	65	10	24	80	13	6.6
Si-10Ta-600	65	6.9	28	70	11	19	79	14	6.9
Si-10Ta-500	70	7.4	23	66	16	18	79	15	6.3
Si-10Ta-400	67	9.3	24	65	16	18	84	10	5.3

## Data Availability

Not applicable.

## Supplementary Materials

The following supporting information can be downloaded at: https://www.mdpi.com/article/10.3390/membranes12090889/s1, Figure S1: SEM surface micrographs of each membrane surface, Figure S2: XANES XRF spectra using a fluorescence detector on membrane Si-10Ta-650, Table S1: Raw peak fitting results for the XPS of all tested membranes. Peak fitting was performed on the data with no manipulation, and peaks were not shifted based on the C1s position. The C1s peak was undetected for all membranes after the 30 s sputter, so no position is known to account for localized charging.
