# Supplementary material for: Synthesis and Characterization of Silica–Tantala Microporous Membranes for Gas Separations Fabricated Using Chemical Vapor Deposition
Source: Membranes (Basel). 2022 Sep 16;12(9):889. doi: 10.3390/membranes12090889 (PMC9503561; doi:10.3390/membranes12090889)
Supplement: Supplementary file 1 [file membranes-12-00889-s001.zip › membranes-1900200-supplementary.pdf]

Supplementary Information

Synthesis and Characterization of Silica-Tantala Microporous Membranes for Gas Separations Fabricated Using Chemical Vapor Deposition

Sean-Thomas B. Lundin [1,2], Hongsheng Wang [1] and S. Ted Oyama [1,3,4,*]

[1] Department of Chemical System Engineering, The University of Tokyo, 7-3-1 Hongo, Bunkyo-ku, Tokyo 113-8556, Japan
[2] National Institute of Advanced Industrial Science and Technology (AIST), Research Institute of Chemical Process Technology, 4-2-1 Nigatake, Miyagino-ku, Sendai 983-8551, Japan
[3] College of Chemical Engineering, Fuzhou University, Fuzhou 350116, China
[4] Department of Chemical Engineering, Virginia Tech, Blacksburg, VA 24061, USA
* Correspondence: oyama@vt.edu; Tel.: +81-3-5841-0712

S.1. SEM surface micrographs

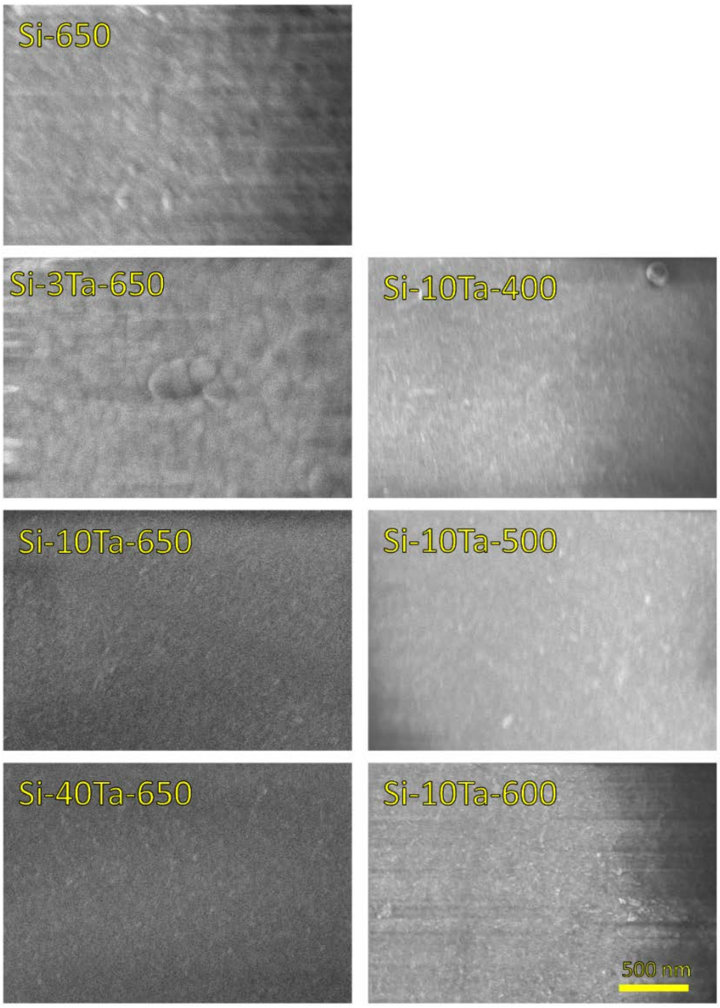

Figure S1. SEM surface micrographs of each membrane surface.

S.2. XANES spectra

The X-ray absorption near edge spectra (XANES) measurements in the Ta L_{III}-edge region (9841 eV – 9946 eV) were performed at the Institute of Materials Structure Science, High-Energy Accelerator Research Organization (KEK-IMSS) in Tsukuba, Japan. Either fluorescence or transmission mode was used. Transmittance measurements were conducted at the Photon Factory (PF) beamline BL9C, using X-rays generated at 2.5 GeV with a beam current of 450 mA; the monochromator was detuned to eliminate higher harmonics included in the incident beam and the measurements were recorded in a step scanning mode using ionization chambers filled with 100% N_2 for the detectors of the incident X-ray beam (I_0) and 15% Ar in N_2 for the transmitted beam (I). Fluorescence measurements were conducted at the Photon Factory Advanced Ring (PF-AR) beamline NW10A, using X-rays generated at 6.5 GeV and 50 mA, the measurements were recorded in a step scanning mode using ionization chambers filled with 100% N_2 for the detector of the incident X-ray beam (I_0) and a multi-channel Ge solid state detector (SSD) for that of the fluorescence beam (I). Scan times on the samples were 60 s using transmittance modes or 17.5 min per scan for the fluorescence mode. Samples were prepared by scraping the surface of the membrane support using a spatula and collecting the silica particles. All measurements were conducted at room temperature and open to atmosphere.

The XANES analysis was not able to detect any Ta within the samples. The XANES XRF spectra of Si-10Ta-650 are presented in Figure S1, which shows that the spectra taken before and after the Ta L3-edge are similar, suggesting no Ta within this sample. A similar test was conducted using a sample of Si-40Ta-650 using transmittance mode, but no change in the spectra before and after the expected Ta region could be detected. These results suggest the deposition of tantala was exceedingly slow compared to silica during CVD synthesis, consistent with EDS and XPS results.

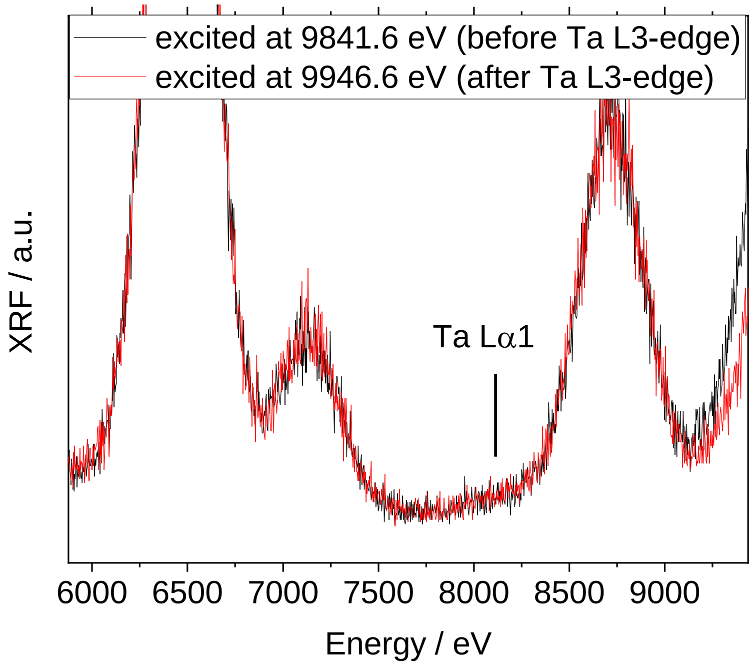

Figure S1. XANES XRF spectra using a fluorescence detector on membrane Si-10Ta-650.

S.3. XPS raw data

Table S1. Raw peak fitting results for the XPS of all tested membranes. Peak fitting was performed on the data with no manipulation, and peaks have not been shifted based on the C1s position. The C1s peak was undetected for all membranes after the 30 s sputter, so no position is known to account for localized charging.

Element Peak	Membrane ID	Before Sputtering				After Sputtering			
		Position	FWHM	Height	Area	Position	FWHM	Height	Area
C1s	Si-650	284.1	1.60	1295	2333	-	-	-	-
	Si-3Ta-650	283.9	1.39	518	1100	-	-	-	-
	Si-10Ta-650	284.0	1.53	1588	3534	-	-	-	-
	Si-40Ta-650	284.0	1.22	616	1188	-	-	-	-
	Si-10Ta-600	284.0	1.54	691	1375	-	-	-	-
	Si-10Ta-500	284.1	1.55	256	523	-	-	-	-
	Si-10Ta-400	284.2	1.54	296	854	-	-	-	-
O1s	Si-650	532.2	1.65	11064	19438	532.6	1.87	11861	23609
	Si-3Ta-650	532.0	1.54	14322	23440	532.6	1.74	14942	27814
	Si-10Ta-650	531.3	1.91	8487	17234	532.1	2.11	10780	24199
	Si-40Ta-650	532.1	1.42	16531	24922	532.4	1.98	13145	27751
	Si-10Ta-600	531.9	1.62	10316	17802	532.3	2.02	10852	23317
	Si-10Ta-500	532.0	1.70	13494	24386	532.4	2.16	12176	28031
	Si-10Ta-400	531.9	1.79	11156	21298	532.3	2.11	10866	24418
Al2p	Si-650	-	-	-	-	75.0	1.47	35	55
	Si-3Ta-650	-	-	-	-	-	-	-	-
	Si-10Ta-650	74.0	1.62	759	1312	75.0	1.69	1372	2466
	Si-40Ta-650	74.4	1.68	334	598	75.2	1.69	840	1510
	Si-10Ta-600	74.2	1.56	358	594	74.6	2.2	569	1332
	Si-10Ta-500	74.2	1.36	844	1218	75.3	1.67	1547	2757
	Si-10Ta-400	74.1	1.53	736	1201	75.2	1.64	1461	2552
Si2p	Si-650	102.9	1.99	2577	5470	103.4	1.91	3273	6820
	Si-3Ta-650	102.7	1.67	3518	6357	103.4	1.81	3868	7449
	Si-10Ta-650	102.3	1.73	1443	2658	103.0	2.03	1556	3364
	Si-40Ta-650	102.8	1.65	3332	5886	103.2	1.96	2489	5206
	Si-10Ta-600	102.7	1.68	2302	4215	103.2	1.91	1930	3923
	Si-10Ta-500	102.8	1.66	2739	5016	103.3	1.9	1803	3732
	Si-10Ta-400	102.7	1.71	2136	4076	103.2	1.85	1420	2801
